# The current status of renal cell carcinoma and prostate carcinoma grading

**DOI:** 10.1590/S1677-5538.IBJU.2018.06.01

**Published:** 2018

**Authors:** Brett Delahunt, Lars Egevad, John Yaxley, Hemamali Samaratunga

**Affiliations:** 1 Department of Pathology and Molecular Medicine, Wellington School of Medicine and Health Sciences, Wellington, New Zealand; 2 Department of Pathology, Karolinska Institute, Stockholm, Sweden; 3 Department of Oncology-Pathology, Karolinska Institute, Stockholm, Sweden; 4 Wesley Hospital, Brisbane, Queensland, Australia; 5 University of Queensland School of Medicine, Brisbane, Queensland, Australia; 6 Aquesta Uropathology, Brisbane, Queensland, Australia

## INTRODUCTION

Grading is an important prognostic indicator for tumors and for most malignancies provides information additional to staging. As with staging, grading criteria for individual tumors are subject to change, with developments reflecting contemporary advances in our understanding of the behavior of tumors. In the field of urological pathology, the grading classifications most commonly utilized for both renal cell carcinoma (RCC) and prostate adenocarcinoma (PCa) have undergone radical change. This evolution has, most recently, led to the establishment of novel grading systems for both of these tumors, under the auspices of the International Society of Urological Pathology (ISUP) (1, 2). The release of the Fourth Edition of the World Health Organization (WHO) Bluebook on the Classification of Tumours of the Urinary Tract and Male Genital Organs in 2016 (3), followed on from the development of these contemporary grading classifications. In this publication these novel classifications, relating to the two most common morphotypes of RCC and for PCa, were endorsed for international implementation. Subsequently both grading classifications have been incorporated into the reporting datasets issued by the International Collaboration on Cancer Reporting (4, 5).

### Renal cell carcinoma grading

Numerous grading systems for renal malignancies have been proposed with validation studies often providing conflicting results (6). While a variety of grading parameters for RCC have been proposed the concept of nuclear grading for these tumors was established 50 years ago by Myers et al. This was followed three years later by the publication of a comprehensive nuclear grading system by Skinner; however, in 1972 this was modified into the four tier grading system of Fuhrman, Lasky and Limas (7- 9). Although Fuhrman grading has been utilized internationally for almost 50 years, it is now widely recognized that the system is hampered by uncertainties relating to reproducibility and its validity as a prognostic marker (10). Among the criticisms applied to Fuhrman grading is the fact that it relies on the simultaneous assessment of nuclear shape and size, as well as nucleolar prominence. This implies that these parameters increase in parallel with increasing grade. Unfortunately, in reality, these parameters are discordant in over 20% of clear cell RCC and as a consequence Fuhrman grading cannot be applied to these cases (10, 11).

These issues were addressed at the Consensus Conference of ISUP convened in Vancouver in 2012 (1). The meeting adopted a grading system derived from assessment of series of clear cell, papillary and chromophobe RCCs (12-14). These studies demonstrated that for clear cell RCC nuclear size and nucleolar prominence, based on the high power field showing the highest grade features, was significantly associated with outcome. For papillary RCC only nucleolar prominence correlated with outcome, while for chromophobe RCC not one of the three parameters of the Fuhrman grading system was found to have prognostic significance. Informed by these findings the novel grading system adopted by the ISUP was based upon nucleolar prominence (1). In this grading classification grade 1 tumors show inconspicuous to small basophilic nucleoli visible at 400x magnification; In grade 2 tumours nucleoli are eosinophilic and prominent at 400x magnification, but inconspicuous at 100x magnification. Grade 3 tumors have nucleoli clearly seen as prominent at 100x magnification. Features required for tumours to be classified as grade 4 are any of the following: 1. sarcomatoid morphology (sarcoma-like mesenchymal to epithelial translocation), 2. rhabdoid morphology, 3. extreme nuclear pleomorphism and 4. anaplastic tumour giant cells. The grading system was formally recommended by the ISUP for both clear cell and papillary RCC. In the absence of evidence that grading was of prognostic significance for chromophobe RCC, it was agreed that this tumor type should not be graded (1). The literature relating to the ISUP grading system for RCC was considered at the 2014 meeting of the WHO Renal Tumour Classification Panel. At this meeting the grading system was endorsed by the WHO and was incorporated into the fourth edition WHO renal tumour classification being designated the WHO/ISUP Grading System (3).

While the WHO/ISUP grading system is applicable only to clear cell and papillary RCC it was agreed that it may also be utilized for descriptive purposes for other morphotypes of RCC (4). If grading is applied to morphotypes other than clear cell and papillary RRC, it is recommended that it be clearly stated in the pathology report that grading is provided for descriptive purposes only and that grading has not been validated for any specific type of renal cell neoplasia, other than clear cell and papillary RCC (15).

WHO/ISUP grading has been validated in a number of studies for both clear cell and papillary RCC (11, 16-18). An interesting, but perhaps not surprising feature of the new grading system, is that when WHO/ISUP graded cases of both clear cell and papillary RCC were compared to those cases in the same series for which Fuhrman grading could be applied, there was a down-grading, with an increase in cases assigned into WHO/ISUP grades 1 and 2 (11, 18). This is a reflection of the fact that for WHO/ISUP grades 2 and 3 tumors, the degree of nucleolar prominence required is greater than that needed to assign tumors into grades 2 and 3 of the Fuhrman system.

While the WHO/ISUP grading classification provides prognostic information for clear cell RCC, studies have suggested that this may be improved if the presence/absence of tumor-related necrosis is incorporated into a revised grading system (16, 19, 20). This specific form of necrosis must be differentiated from thrombo-embolic coagulative necrosis, being characterized by loss of architecture and has been shown to have prognostic significance independent of both tumor grade and TNM staging category (10). In a modified grading system, in which WHO/ISUP grade was sub-stratified on the basis of absence/presence of tumor-related necrosis it was shown that WHO/ ISUP grade 2 tumors with necrosis had an outcome similar to WHO/ISUP grade 3 tumors without necrosis and similarly that WHO/ISUP grade 3 tumors with necrosis had an outcome similar to grade 4 tumours without necrosis (16, 19, 20). These results suggest that this modified grading system has an enhanced positive predictive value when compared to WHO/ISUP grading alone.

### Prostate adenocarcinoma grading

Numerous grading systems have been proposed for PCa, of which the system that achieved worldwide acceptance for decades has been that of Donald Gleason (20, 21). Since the introduction of the Gleason system in 1966, a number of modifications have been proposed, with the latest being in 2016 (22). Gleason based his grading purely on architecture without taking cytological atypia into consideration. Five grades were created from the lowest grade of 1 to the highest grade of 5. The dominant and subdominant grades were added to create the Gleason score of 2 to 10. In Gleason's series, which pre-dated the introduction of thin core needle biopsy of the prostate, 88.5% of patients presented with extra-prostatic disease, with 36% having metastases (23). This situation changed dramatically with the introduction of prostate specific antigen (PSA) testing in 1994 (24). Since then, the number of patients presenting with metastatic PCa has decreased markedly (25). During this time, many other aspects of PCa diagnosis and treatment have also evolved. It also became apparent that not all tumor patterns in the Gleason system were classified correctly and in particular the designation of cribriform glands as grades 2 and 3, as well as single cells and solid cords and masses as grade 3, was inappropriate as these patterns are now recognized as features of high-grade disease.

While initial modifications to the Gleason system were made by Gleason himself in 1974 and 1977 (26, 27), other major changes were introduced in 2005 at a Consensus Conference convened by the International Society of Urological Pathology (28). At this meeting, it was decided that Gleason grade 1 cancer represented adenosis, and therefore should not be diagnosed irrespective of the type of prostate specimen. It was also decided that grade 2 should be diagnosed rarely, if ever, in needle biopsies and that cribriform glands were indicative of, at least, grade 3 tumor. Cribriform cancer, which consisted of small round uniform glands with regular round lumina, were considered grade 3, while all other patterns of cribriform glands were considered grade 4. As recommended by Gleason, it was agreed that the presence of comedonecrosis was a feature of grade 5, while Grade 4 was expanded to include poorly formed acini. There was agreement that the grading of all variants of PCa, other than mucinous tumors, should be based upon architecture, ignoring cellular changes. At this meeting, there was no consensus as to how mucinous PCa should be graded (29). A further major change related to scoring of needle biopsies containing a higher grade tertiary pattern. Here it was decided that the Gleason score should be the sum of most common grade (primary grade) and the highest grade present. This change was recommended to avoid sample bias inherent in needle biopsies leading to apparent downgrading in contrast to grading of radical prostatectomy specimens, where the entire tumor is available for assessment. Subsequent studies have confirmed the validity of these modifications in improving the predictive value of needle biopsy grading in relation to grading of radical prostatectomy specimens, as well as biochemical recurrence free survival and overall survival rates (30-32).

More recent studies have highlighted difficulties in differentiating grade 3 cribriform PCa from cribriform cancers with a grade 4 morphology and it is now recognized that all cribriform PCa has a uniformally unfavourable prognosis (33, 34). A further issue related to the assignment of an overall grade to a cancer as in the 2005 modified system Gleason scores ranged from 6 to 10. From a management viewpoint it was apparent that there was value in differentiating cases into prognostic categories such as low, intermediate and high-grade. In 1977, Gleason suggested grouping scores of 2-3, 4-5, 6, 7 and 8-10 would be clinically valid (27, 35). While more recently others have proposed a variety of different combinations of Gleason scores to produce valid prognostic groups (22, 36-39).

To formulate changes to PCa grading, the ISUP convened a further Consensus Conference in 2014 attended by 82 experts from 19 countries (2). At this conference additional amendments to the 2005 Gleason grading criteria were recommended. In particular all cribriform and glomeruloid patterns of tumour were classified as Gleason grade 4 and it was agreed that mucinous adenocarcinoma grading should be based upon the underlying architecture. It was also decided that intraductal carcinoma should not be assigned a grade. Five prognostic categories labelled ISUP grades were created. Gleason score 3+3 were reclassified as Grade 1, Gleason score 3+4 as Grade 2, Gleason score 4+3 as Grade 3, Gleason score 8 (4+4, 3+5, 5+3) as Grade 4, and Gleason scores 9 and 10 as Grade 5. It was decided that, if present, a higher tertiary pattern would continue to be applied to grading as the secondary pattern in needle biopsies. In contrast, there was no agreement as to how tertiary patterns should be dealt with in radical prostatectomy specimens, which means that ISUP grading cannot be strictly applied to these specimens. Several studies have subsequently validated this new ISUP grading system with respect to patient outcome (40-42).

It has subsequently been suggested that tertiary patterns in radical prostatectomy specimens should be treated as a high-grade component in the Gleason score if > 5% of tumor volume. This recommendation has not been validated and was not a consensus decision of the 2014 ISUP meeting. Other recent recommendations are that the percentage of pattern 4 and 5 in both needle biopsies and radical prostatectomies should be recorded as they appear to provide additional prognostic information (43, 44). Currently, the optimum method for evaluating the volume of a higher-grade PCa, is uncertain at it could be based on measurement of surface area or length of the biopsy core. There is also debate as to whether percent pattern 4/5 should be reported for individual cores or for the entire case. Since the introduction of ISUP grading another issue that has been highlighted is the validity of grouping of Gleason score 4+4, 3+5 and 5+3 tumors into ISUP grade 4 category, as subsequent studies have suggested that each of these differ in outcome (45). While the introduction of the ISUP grading system for prostate core biopsies has resulted in significant improvements in outcome prediction for PCa, it remains a system in evolution.

## References

[1] Delahunt B, Cheville JC, Martignoni G, Humphrey PA, Magi-Galluzzi C, McKenny J (2013). The International Society of Urological pathology (ISUP) grading system for renal cell carcinoma and other prognostic factors. Am J Surg Pathol.

[2] Epstein JI, Egevad L, Amin MB, Delahunt B, Srigley JR, Humphrey PA (2016). The 2014 International Society of Urological Pathology (ISUP) Consensus Conference on Gleason Grading of Prostatic Carcinoma Definition of Grading Patterns and Proposal for a New Grading System. Am J Surg Pathol.

[3] Humphrey PA, Moch H, Reuter VE, Ulbright TM, World Health Organization (WHO) (2016). Classification of tumours. Pathology and genetics of the urinary system and male genital organs.

[4] Delahunt B, Srigley JR, Amin MB, Billis A, Camparo P, Evans AJ (2017). Invasive Carcinoma of Renal Tubular Origin, Histopathology Reporting Guide.

[5] Egevad L, Kench JG, Delahunt B, Humphrey PA, Kristiansen G, Oxley JD (2017). Prostate Cancer, Radical Prostatectomy, Histopathology Reporting Guide.

[6] Delahunt B (2009). Advances and controversies in grading and staging of renal cell carcinoma. Mod Pathol.

[7] Myers GH, Fehrenbaker LG, Kelalis PP (1968). Prognostic significance of renal vein invasion by hypernephroma. J Urol.

[8] Skinner DG, Colvin RB, Vermillion CD, Pfister RC, Leadbetter WF (1971). Diagnosis and management of renal cell carcinoma. A clinical and pathologic study of 309 cases. Cancer.

[9] Fuhrman SA, Lasky LC, Limas C (1982). Prognostic significance of morphologic parameters in renal cell carcinoma. Am J Surg Pathol.

[10] Delahunt B, Egevad L, Samaratunga H (2018). Grading of renal cell carcinoma. Histopathology.

[11] Dagher J, Delahunt B, Rioux-Leclercq N, Egevad L, Srigley JR, Coughlin G (2016). Clear cell renal cell carcinoma: validation of World Health Organization/International Society of Urological Pathology grading. Histopathology.

[12] Delahunt B, Sika-Paotonu, Bethwaite PB, Jordan TW, Magi-Galluzzi C, Zhou M (2011). Grading of clear cell renal cell carcinoma should be based on nucleolar prominence. Am J Surg Pathol.

[13] Sika-Paotonu D, Bethwaite PB, McCredie MRE, Jordan TW, Delahunt B (2006). Nucleolar grade but not Fuhrman grade is applicable to papillary renal cell carcinoma. Am J Surg Pathol.

[14] Delahunt B, Sika-Paotonu D, Bethwaite PB, McCredie MRE, Martignoni G, Jordan TW (2007). Fuhrman grading is not appropriate for chromophobe renal cell carcinoma. Am J Surg Pathol.

[15] Delahunt B, McKenney JK, Lohse CM, Leibovich BC, Thompson RH, Boorjian SA (2013). A novel grading system for renal cell carcinoma incorporating tumor necrosis. Am J Surg Pathol.

[16] Sukov WR, Lohse CM, Leibovich BC, Thompson RH, Cheville JC (2012). Clinical and pathological features associated with prognosis in patients with papillary renal cell carcinoma. J Urol.

[17] Cornejo KM, Dong F, Zhou AG, Wu C-L, Young RH, Braaten K (2015). Papillary renal cell carcinoma: correlation of tumor grade and histologic characteristics with clinical outcome. Hum Pathol.

[18] Khor L-Y, Dhakal HP, Jia X, Reynolds JP, McKenney JK, Rini BI (2016). Tumor necrosis adds prognostically significant information to grade in clear cell renal cell carcinoma. A study of 842 consecutive cases from a single institution. Am J Surg Pathol.

[19] Dagher J, Delahunt B, Rioux-Leclercq R, Egevad L, Coughlin G, Dunglison N (2018). Assessment of tumour associated necrosis provides prognostic information additional to WHO/ISUP grading for clear cell renal cell carcinoma. Histopathology.

[20] Delahunt B, Srigley JR, Lamb DS (2009). Gleason grading: consensus and controversy. Pathology.

[21] Gleason DF (1966). Classification of prostatic carcinomas. Cancer Chemother Rep.

[22] Srigley JR, Delahunt B, Egevad L, Samaratunga H, Yaxley J, Evans AJ (2016). One is the new six: the International Society of Urological Pathology (ISUP) patient-focused approach to Gleason grading. CUAJ.

[23] Bailar JC, Mellinger GT, Gleason DF (1966). Survival rates of patients with prostatic cancer, tumor stage, and differentiation - preliminary report. Cancer Chemother Rep.

[24] Ruckle HC, Klee GG, Oesterling JE (1994). Prostate-specific antigen: critical issues for the practicing physician. Mayo Clin Proc.

[25] Scosyrev E, Wu G, Mohile S, Messing EM (2012). Prostate-specific antigen screening for prostate cancer and the risk of overt metastatic disease at presentation: analysis of trends over time. Cancer.

[26] Gleason DF, Mellinger GT (1974). Prediction of prognosis for prostatic adenocarcinoma by combined histological grading and clinical staging. J Urol.

[27] Gleason DF, Tannenbaum M Histological grading and clinical staging of prostatic carcinoma. Urologic Pathology: The Prostate.

[28] Delahunt B, Miller RJ, Srigley JR, Evans AJ, Samaratunga H (2012). Gleason grading: past, present and future. Histopathology.

[29] Epstein JI, Allsbrook WC, Amin MB, Egevad LL, ISUP Grading Committee (2005). The 2005 International Society of Urological Pathology (ISUP) Consensus Conference on Gleason Grading of Prostatic Carcinoma. Am J Surg Pathol.

[30] Ozok HU, Sagnak L, Tuygun COktay M, Karakoyunlu N, Ersoy H (2010). Will the modification of the Gleason grading system affect the urology practice?. Int J Surg Pathol.

[31] Tsivian M, Sun L, Mouraviev VMadden JF, Mayes JM, Moul JW (2009). Changes in Gleason score grading and their effect in predicting outcome after radical prostatectomy. Urology.

[32] Uemura H, Hoshino K, Sasaki TMiyoshi Y, Ishiguro H, Inayama Y (2009). Usefulness of the 2005 International Society of UrologicPathology Gleason grading system in prostate biopsy and radical prostatectomy specimens. BJU Int.

[33] Sarbay BC, Kir G, Topal CS, Gumus E (2014). Significance of the cribriform pattern in prostatic adenocarcinomas. Pathol Res Pract.

[34] Kir G, Sarbay BC, Gumus E, Topal CS (2014). The association of the cribriform pattern with outcome for prostatic adenocarcinomas. Pathol Res Pract.

[35] Gleason DF (1992). Histologic grading of prostate cancer: a perspective. Hum Pathol.

[36] Samaratunga H, Delahunt B, Egevad L, Srigley JR, Yaxley J (2017). The Evolution of Gleason Grading of Prostate Cancer. J Diagn Pathol.

[37] Donohue JF, Bianco FJ, Kuroiwa K, Vickers AJ, Wheeler TM, Scardino PT (2006). Poorly differentiated prostate cancer treated with radical prostatectomy: long-term outcome and incidence of pathological downgrading. J Urol.

[38] Tolonen TT, Kujala PM, Tammela TLJ, Tuominen VJ, Isola JJ, Visakorpi T (2011). Overall and worst Gleason scores are equally good predictors of prostate cancer progression. BMC Urology.

[39] Eifler JB, Feng Z, Lin BM, Partin MT, Humphreys EB, Han M (2013). An updated prostate cancer staging nomogram (Partin tables) based on cases from 2006 to 2011. BJU Int.

[40] Samaratunga H, Delahunt B, Gianduzzo T, Coughlin G, Duffy D, LeFevre I (2015). The prognostic significance of the 2014 International Society of Urological Pathology (ISUP) grading system for prostate cancer. Pathology.

[41] Delahunt B, Egevad L, Srigley JR, Steigler A, Murray JD, Atkinson C (2015). Validation of International Society of Urological Pathology (ISUP) grading for prostatic adenocarcinoma in thin core biopsies using TROG 03.04 ‘RADAR’ trial clinical data. Pathology.

[42] Berney DM, Beltran L, Fisher G, North BV, Greenberg D, Moller H (2016). Validation of a contemporary prostate cancer grading system using cancer death as outcome. Br J Cancer.

[43] Kir G, Seneldir H, Gumus E (2016). Outcomes of Gleason score 3.4 7 prostate cancer with minimal amounts (<6%) vs ≥6% of Gleason pattern 4 tissue in needle biopsy specimens. Ann Diagn Pathol.

[44] Sauter G, Steurer S, Clauditz TS, Krech T, Wittmer C, Lutz F (2016). Clinical utility of quantitative Gleason grading in prostate biopsies and prostatectomy specimens. Eur Urol.

[45] Egevad L, Delahunt B, Evans A, Grignon DJ, Kench JG, Kristiansen G (2016). International Society of Urological Pathology (ISUP) grading of prostate cancer. Am J Surg Pathol.

